# Prioritizing antiviral drugs against SARS-CoV-2 by integrating viral complete genome sequences and drug chemical structures

**DOI:** 10.1038/s41598-021-83737-5

**Published:** 2021-03-18

**Authors:** Lihong Peng, Ling Shen, Junlin Xu, Xiongfei Tian, Fuxing Liu, Juanjuan Wang, Geng Tian, Jialiang Yang, Liqian Zhou

**Affiliations:** 1 School of Computer Science, Hunan University of Technology, Zhuzhou, 412007 China; 2 College of Computer Science and Electronic Engineering, Hunan University, Changsha, 410082 China; 3 Geneis (Beijing) Co. Ltd., Beijing, 100102 China

**Keywords:** Computational biology and bioinformatics, Drug discovery, Computer science, Information technology

## Abstract

The outbreak of a novel febrile respiratory disease called COVID-19, caused by a newfound coronavirus SARS-CoV-2, has brought a worldwide attention. Prioritizing approved drugs is critical for quick clinical trials against COVID-19. In this study, we first manually curated three Virus-Drug Association (VDA) datasets. By incorporating VDAs with the similarity between drugs and that between viruses, we constructed a heterogeneous Virus-Drug network. A novel Random Walk with Restart method (VDA-RWR) was then developed to identify possible VDAs related to SARS-CoV-2. We compared VDA-RWR with three state-of-the-art association prediction models based on fivefold cross-validations (CVs) on viruses, drugs and virus-drug associations on three datasets. VDA-RWR obtained the best AUCs for the three fivefold CVs, significantly outperforming other methods. We found two small molecules coming together on the three datasets, that is, remdesivir and ribavirin. These two chemical agents have higher molecular binding energies of − 7.0 kcal/mol and − 6.59 kcal/mol with the domain bound structure of the human receptor angiotensin converting enzyme 2 (ACE2) and the SARS-CoV-2 spike protein, respectively. Interestingly, for the first time, experimental results suggested that navitoclax could be potentially applied to stop SARS-CoV-2 and remains to further validation.

## Introduction

In late December, 2019, there was an outbreak of a novel febrile respiratory illness (COVID-19) in Wuhan, Hubei in China^1,2^. The illness was caused by a novel coronavirus named SARS-CoV-2 by the World Health Organization (WHO) and can transmit from human to human^2^. As of 10 a.m. Cest time on October, 18, 2020, 40,118,333 cases of SARS-CoV-2 infection and 1,114,749 cases of SARS-CoV-2-caused death have been confirmed around the world^3^. From February, 2020, WHO is seeking U.S. $675 million for COVID-19 preparedness to prevent human to human transmission^4^.

SARS-CoV-2 is a new human-infecting single-stranded RNA virus^2^. It is very similar to two coronaviruses: severe acute respiratory syndrome (SARS) coronavirus (SARS-CoV) and Middle East respiratory syndrome (MERS) coronavirus (MERS-CoV). In November, 2002, SARS first emerged in Guangdong, China, and resulted in 8,098 infection cases with a fatality rate of 9.6%^1^. In September, 2012, MERS was first found in humans in the Middle East and resulted in 2,465 laboratory-confirmed cases of infection with a fatality rate 34.4%^5^.

As SARS-CoV-2 is an emerging virus, no specific antiviral treatment has been developed^6^. Therefore, finding effective drug treatment options is urgently needed for combating SARS-CoV-2^7^. However, it seems unrealistic to test new drugs targeting SARS-CoV-2 within such limited time^8^. An efficient method is to screen possible drugs from available public data repositories containing FDA-approved compounds^7,9^. Under such situation, computational methods could be chosen to identify special antiviral drug candidates^10–12^.

Although little is known about SARS-CoV-2, its complete genome sequence suggests strong homology with SARS-CoV^13^. To identify possible antiviral drugs, in this study, we investigated the relationship between the complete genome sequences of viruses similar to SARS-CoV-2, the chemical structures of drugs, and Virus-Drug Association (VDA) network topology. We then developed a novel Random Walk with Restart method (VDA-RWR) to find possible VDAs related to SARS-CoV-2 by integrating the genome sequences and the chemical structures into a unified framework. We compared VDA-RWR with NGRHMDA^14^, SMiR-NBI^15^ and LRLSHMDA^16^. These three methods were applied to biological association prediction in other application areas and obtained better prediction performance. We found that remdesivir and ribavirin come together on three datasets.

Molecular docking is a key bioinformatics modeling tool for drug discovery and used to predict the “best-fit” intermolecular binding between a small molecule and a target or two proteins at the atomic level. It characterizes the behavior of ligands in the binding sites of target proteins as well as elucidates fundamental biochemical processes^17^. The docking process comprises two basic steps: predicting conformation, position, and orientation of ligands within the binding sites and ranking these conformations based on the binding affinity^18^. We used AutoDock^19^, a molecular docking software, to measure the molecular activities of the predicted two compounds (remdesivir and ribavirin) binding to the SARS-CoV-2 spike protein/human receptor angiotensin converting enzyme 2 (ACE2). The docking showed that remdesivir and ribavirin have higher binding energies of − 7.0 kcal/mol and − 6.59 kcal/mol with the structure of the spike protein receptor-binding domain bound to the ACE2 receptor, respectively.

## Results

### Experimental settings

In this section, we conducted extensive experiments to investigate the performance of our proposed VDA-RWR method. For the VDA matrix ${Y}_{n\times m}$ from $n$ viruses and $m$ drugs, fivefold Cross-Validations (CVs) were performed under the following three different experimental settings.

- Fivefold Cross Validation 1 (CV1): CV on viruses, that is, random rows in $Y$ (i.e., viruses) were selected for testing.
- Fivefold Cross Validation 2 (CV2): CV on drugs, that is, random columns in $Y$ (i.e., drugs) were selected for testing.
- Fivefold Cross Validation 3 (CV3): CV on virus-drug pairs, that is, random entries in $Y$ (i.e., virus-drug pairs) were selected for testing.

Under CV1, in each round, 80% of rows in $Y$ were used as training set and the remaining 20% of rows were used as test set. Under CV2, in each round, 80% of columns in $Y$ were used as training set and the remaining 20% of columns were used as test set. Under CV3, in each round, 80% of entries in $Y$ were used as training set and the remaining 20% of entries were test set. These three settings CV1, CV2, and CV3 specially refer to potential VDA identification for (1) new viruses (especially for SARS-CoV-2), (2) new drugs, and (3) new virus-drug pairs, respectively.

Parameters $r$, $\mu$, and $\alpha$ denote the global restart rate, the transition probability, and the weight between the virus network and the drug network, respectively. For these three parameters, we performed cross validations on the training set to find the optimal values. In addition, the iteration stopped when ${\left\| {p}_{t+1}-{p}_{t} \right\|}_{2}\le 1e-11$. SMiR-NBI need not set the parameters. For the parameters in NGRHMDA and LRLSHMDA, we conducted grid search to find the optimal values. The detailed settings are shown on Table 1.

**Table 1:** The optimal values of parameters in VDA-RWR, NGRHMDA and LRLSHMDA.

Method	Dataset 1	Dataset 2	Dataset 3
NGRHMDA	α = 0.4, β = 0.8	α = 0.6, β = 0.9	α = 0.9, β = 0.9
LRLSHMDA	ηM = 0.9, ηD = 0.3	ηM = 0.8, ηD = 0.1	ηM = 0.6, ηD = 0.1
VDA-RWR	r = 0.7, μ = 0.9, α = 0.5	r = 0.5, μ = 0.9, α = 0.9	r = 0.7, μ = 0.9, α = 0.9

### Evaluation metrics

Sensitivity, specificity, F1 score, accuracy and AUC were widely applied to evaluate the proposed methods. Sensitivity denotes the ratio of correctly predicted positive VDAs to all positive VDAs. Specificity is the ratio of correctly predicted negative VDAs to all negative VDAs (all the unknown associations were labeled as negative). F1 score denotes the harmonic mean of recall and precision. Accuracy represents the ratio of correctly predicted positive and negative VDAs to all positive and negative VDAs. We used these five metrics to evaluate the performance of VDA-RWR. They were defined as follows:

1 $$Sensitivity=\frac{TP}{TP+FN}$$

2 $$Specificity=\frac{TN}{TN+FP}$$

3 $$Accuracy=\frac{TP+TN}{TP+TN+FP+FN}$$

4 $$F1 score=\frac{2TP}{2TP+FP+FN}$$

where $TP$, $FP$, $TN$ and $FN$ represent true positive, false positive, true negative and false negative, respectively.

AUC is the average area under the receiver operating characteristics (ROC) curve. The curve can be plotted by the ratio of True Positive Rate (TPR) to False Positive Rate (FPR) according to different thresholds. TPR and FPR are defined via Eqs. (4–5).

5 $$TPR=\frac{TP}{TP+FN}=\frac{TP}{T}$$

6 $$FPR=\frac{FP}{FP+TN}=\frac{FP}{F}$$

For these five evaluation metrics, higher values represent better performance.

### Performance evaluation under three fivefold CVs

We compared VDA-RWR with NGRHMDA^14^, SMiR-NBI^15^ and LRLSHMDA^16^. NGRHMDA was presented to find potential microbe-disease associations by integrating neighbor-based collaborative filtering and graph-based scoring^14^. SMiR-NBI can comprehensively identify new pharmacogenomic biomarkers by constructing a heterogeneous network connecting genes, drugs, and miRNAs^15^. LRLSHMDA was applied to predict human microbe-disease associations based on Laplacian regularized least squares^16^. These three state-of-the-art approaches obtained good performance in their corresponding applications. We performed these four methods for 100 times on three different fivefold CV settings on three datasets. The final performance was averaged over the five rounds for 100 times. The results are shown in Tables 2, 3, and4. The best results were shown in bold in each column.

**Table 2:** The performance comparison of four methods on three datasets under CV1.

Datasets	Methods	Sensitivity	Specificity	F1 score	Accuracy	AUC
Dataset 1	NGRHMDA	0.7278	0.3991	0.0643	0.4092	0.7026
	SMiR-NBI	**0.8086**	0.2164	0.0366	0.2296	0.5806
	LRLSHMDA	0.1299	0.6171	0.0084	0.6057	0.1844
	VDA-RWR	0.4977	**0.7959**	**0.1055**	**0.7905**	**0.8157**
Dataset 2	NGRHMDA	0.3987	0.5521	0.0329	0.5495	0.4301
	SMiR-NBI	**0.8238**	0.0949	0.0332	0.1087	0.4003
	LRLSHMDA	0.3507	0.4667	0.0179	0.4643	0.3173
	VDA-RWR	0.5106	**0.6832**	**0.0844**	**0.6801**	**0.6932**
Dataset 3	NGRHMDA	0.4435	0.4560	0.0232	0.4563	0.4058
	SMiR-NBI	**0.9124**	0.0459	0.0227	0.0567	0.4092
	LRLSHMDA	0.1801	0.5817	0.0074	0.5766	0.2920
	VDA-RWR	0.5270	**0.7025**	**0.0812**	**0.7006**	**0.7276**

Bold values indicates the best values for the different methods under the same evaluation.

**Table 3:** The performance comparison of four methods on three datasets under CV2.

Datasets	Methods	Sensitivity	Specificity	F1 score	Accuracy	AUC
Dataset 1	NGRHMDA	0.6435	0.6719	0.0850	0.6713	0.8329
	SMiR-NBI	**0.8510**	0.1917	0.0393	0.2064	0.6021
	LRLSHMDA	0.7938	0.5773	0.1122	0.5820	0.8249
	VDA-RWR	0.5070	**0.8932**	**0.1294**	**0.8846**	**0.9182**
Dataset 2	NGRHMDA	0.4867	**0.8027**	0.0719	**0.7967**	0.8017
	SMiR-NBI	**0.9971**	0.0929	0.0404	0.1098	0.7205
	LRLSHMDA	0.7720	0.4166	0.0639	0.4232	0.7334
	VDA-RWR	0.5045	0.7981	**0.0814**	0.7926	**0.8025**
Dataset 3	NGRHMDA	0.4579	0.6785	0.0279	0.6758	0.6772
	SMiR-NBI	**0.9751**	0.0434	0.0243	0.0549	0.5665
	LRLSHMDA	0.7420	0.5264	0.0493	0.5290	0.7468
	VDA-RWR	0.5054	**0.8098**	**0.0628**	**0.8061**	**0.8168**

Bold values indicates the best values for the different methods under the same evaluation.

**Table 4:** The performance comparison of four methods on three datasets under CV3.

Datasets	Methods	Sensitivity	Specificity	F1 score	Accuracy	AUC
Dataset 1	NGRHMDA	0.5783	0.5567	0.0615	0.5572	0.6459
	SMiR-NBI	**0.8331**	0.1936	0.0385	0.2079	0.5723
	LRLSHMDA	0.8034	0.5813	0.1119	0.5863	0.8403
	VDA-RWR	0.4824	**0.7831**	**0.1153**	**0.8278**	**0.8582**
Dataset 2	NGRHMDA	0.4544	0.3562	0.0218	0.3581	0.3011
	SMiR-NBI	**0.8349**	0.0942	0.0336	0.1080	0.4156
	LRLSHMDA	0.7838	0.4840	**0.0733**	0.4896	**0.8248**
	VDA-RWR	0.5022	**0.6643**	0.0574	**0.6613**	0.6675
Dataset 3	NGRHMDA	0.3582	0.4081	0.0119	0.4074	0.2554
	SMiR-NBI	**0.9230**	0.0427	0.0230	0.0536	0.4365
	LRLSHMDA	0.8124	0.5239	0.0552	0.5275	**0.8169**
	VDA-RWR	0.5053	**0.7057**	**0.0556**	**0.7032**	0.7123

Bold values indicates the best values for the different methods under the same evaluation.

On dataset 1 and dataset 3, VDA-RWR outperformed other three methods in terms of specificity, accuracy, F1 score and AUC under three CVs. On dataset 2, although the sensitivity of VDA-RWR was slightly lower, VDA-RWR computed better specificity, accuracy, F1 score and AUC under majority of conditions. The slight difference can be produced by different data structures. AUC is one more important evaluation metric compared to other four measurements. AUC = 0.5 represents random performance and AUC = 1 shows perfect performance. VDA-RWR obtained the best AUCs under majority of conditions. In general, VDA-RWR is proper to discover potential VDAs.

**Figure 1:**
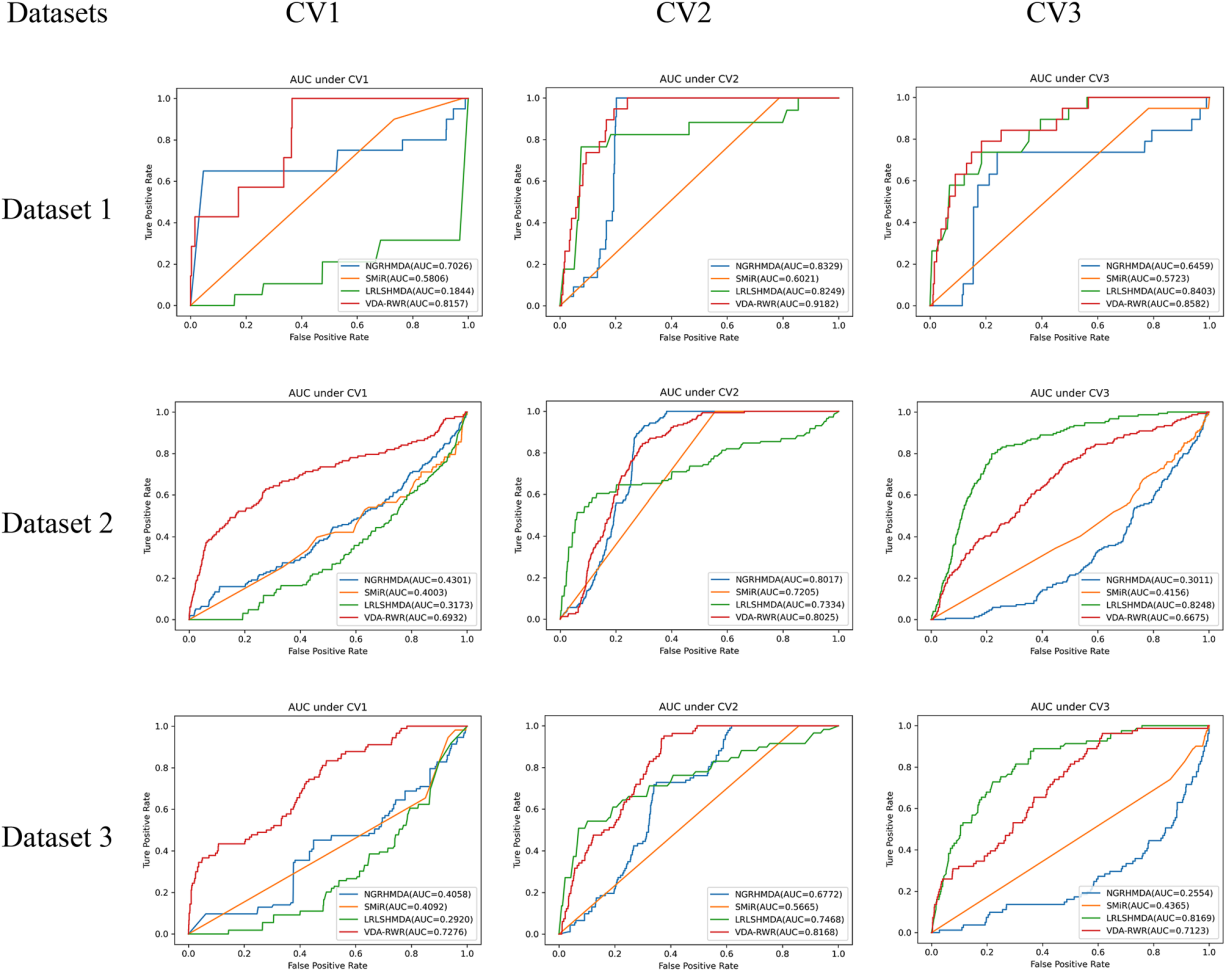
The AUC values of VDA-RWR under different CVs on three datasets.

In addition, under CV1, VDA-RWR computed better specificity, accuracy, F1 score and AUC on the three datasets. This result showed that VDA-RWR can effectively find possible antiviral drugs for new viruses (for example, SARS-CoV-2). Under CV2, VDA-RWR outperformed other three methods in terms of specificity, accuracy, F1 score and AUC on dataset 1 and dataset 3. Although the sensitivity, specificity and accuracy values of VDA-RWR were slightly lower than other individual methods on dataset 2, it obtained the best F1 score and AUC. Thus AUC can identify potential viruses associated with new drugs. Under CV3, VDA-RWR calculated the best specificity, F1 score and accuracy. Figure 1 showed the AUC values of four methods under CV1, CV2, and CV3. The results demonstrated that VDA-RWR obtained relatively higher AUCs under three different CVs. It suggested that VDA-RWR could be used to infer potential VDAs.

### Case study

In this section, we want to find possible drugs for SARS-CoV-2 after verifying the performance of our proposed VDA-RWR method. We predicted the top 10 drugs with the highest association scores with SARS-CoV-2 on three datasets. The results were shown in Tables 5, 6, and 7, respectively. Among the predicted top 10 small molecules associated with SARS-CoV-2 on dataset 1, all drugs were supported by recent works. Among the predicted top 10 chemical agents related to SARS-CoV-2 on dataset 2, there were 9 VDAs validated by current literatures, that is, 90% chemical agents were reported. Among the predicted top 10 antiviral drugs against SARS-CoV-2 on dataset 3, all compounds were validated by recent publications.

**Table 5:** The predicted top 10 drugs associated with SARS-CoV-2.

Rank	Drug	Evidence
1	Remdesivir	PMID: 32020029, 31996494, 32022370, 31971553, 32035018, 32035533, 32036774, 32194944, 32275812, 32145386, 32838064
		https://doi.org/10.1101/2020.01.28.922922
2	Oseltamivir	PMID: 32034637, 32127666
3	Ribavirin	PMID: 32034637, 32127666, 32227493, 26492219, 32771797
4	Zanamivir	PMID: 32511320
5	Presatovir	PMID: 32147628
6	Elvitegravir	PMID: 32147628
7	Zidovudine	PMID: 32568013
8	Emtricitabine	PMID: 32488835
9	Mycophenolic acid	PMID: 32579258
10	Chloroquine	PMID: 32020029, 32145363, 32074550, 32236562

**Table 6:** The predicted top 10 drugs associated with SARS-CoV-2 on dataset 2.

Rank	Drug	Evidence
1	Favipiravir	PMID: 32346491, 32967849, 32972430
2	Remdesivir	PMID: 32020029, 31996494, 32022370, 31971553, 32035018, 32035533, 32036774, 32194944, 32275812, 32145386, 32838064
		https://doi.org/10.1101/2020.01.28.922922
3	Cidofovir	PMID: 32546018
		https://doi.org/10.1007/s10067-020-05133-0
4	Galidesivir	PMID: 32711596
5	Niclosamide	PMID: 32125140, 32221153
6	Mycophenolic acid	PMID: 3257258
7	Itraconazole	https://doi.org/10.22541/au.159467021.16927198
8	Brequinar	PMID: 32426387
9	Navitoclax	Unconfirmed
10	Ribavirin	PMID: 32034637, 32127666, 32227493, 26492219, 32771797

**Table 7:** The predicted top 10 drugs associated with SARS-CoV-2 on dataset 3.

Rank	Drug	Evidence
1	Nitazoxanide	PMID: 32127666, 32568620, 32448490
2	Ribavirin	PMID: 3203637, 32127666, 32227493, 26492219, 32771797
3	Chloroquine	PMID: 32020029, 32145363, 32074550, 32236562
4	Hexachlorophene	PMID: 15950190
5	Camostat	PMID: 32347443
6	Favipiravir	PMID: 32246834
7	Umifenovir	PMID: 32941741
8	Remdesivir	PMID: 32020029, 31996494, 32022370, 31971553, 32035018, 32035533, 32036774, 32194944, 32275812, 32145386, 32838064
		https://doi.org/10.1101/2020.01.28.922922
9	Amantadine	PMID: 32361028
10	Niclosamide	PMID: 32125140, 32221153

The results on Tables 5, 6, and 7 showed that there were two FDA-approved drugs coming together on three datasets, that is, remdesivir and ribavirin. Remdesivir is a small molecular compound undergoing a clinical trial and shows superior antiviral activity against many RNA viruses including orthocoronavirinae, filoviridae, paramyxoviridae, and pneumoviridae^20–22^. Sheahan et al.^17^ presented that it can improve pulmonary function and reduce severe lung pathology in mice. Similar to SARS-CoV-2, both Ebola virus (EBOV) and MERS-CoV may result in severe acute respiratory diseases. And remdesivir has been used as inhibitors of EBOV and MERS-CoV^20,21^. More importantly, an array of works have reported that remdesivir is highly effective in controlling SARS-CoV-2 infection and has been directly applied to the treatment of COVID-19^6,7,9,23–28^. Specially, on October 22, 2020, FDA approved remdesivir for use in adults, pediatric patients with age of 12 years, and older and weighing at least 40 kg^29^. All these results showed that remdesivir may be the best anti-SARS-CoV-2 drug.

Ribavirin is identified as another anti-SARS-CoV-2 drug with a higher association score. Huang et al.^5^ found that 28 of 38 patients treated by ribavirin have been discharged. Zhang et al.^30^ reported that a patient has been treated with antiviral drugs including ribavirin. Therefore, ribavirin may be applied to treat COVID-19 caused by SARS-CoV-2. Interestingly, for the first time, experimental results suggested that navitoclax could be potentially applied to stop SARS-COV-2. Navitoclax has been applied to boost the treatment and basic science of chronic lymphoid leukemia, hematological malignancies, non-Hodgkin's lymphoma, solid tumors, and EGFR activating mutation.

### Molecular docking

The molecular docking between the above two antiviral drugs (remdesivir and ribavirin) and the spike protein and ACE2 are described in Table 8. The results showed that remdesivir and ribavirin have higher binding energies of − 7.0 kcal/mol and − 6.59 kcal/mol with the structure of the spike protein receptor-binding domain bound to the ACE2 receptor, respectively. The subfigure in each circle denotes the residues at the binding site of the spike protein/ACE2 and their corresponding orientations. For example, the amino acids K68 and Q493 were predicted to be the key residues for remdesivir binding to the SARS-CoV-2 spike protein/ACE2 while K353, R403, Q493 and G496 were predicted as the key residues for ribavirin binding to these two target proteins.

**Table 8:** Molecular docking between remdesivir and ribavirin and the SARS-CoV-2 spike (S) protein/ACE2.

Ligand	Molecular formula	Molecular docking	Binding energy (kcal/mol)	Binding sites	Distance(Å)
Remdesivir	C_27_H_3_5N_6_O_8_P		− 7.0	K68	2.0
				Q493	2.3
Ribavirin	C_18_H_26_CIN_3_		− 6.59	K353	2.2
				R403	2.1
				Q493	2.0
				G496	1.9

In Table 8, green denotes the structure of ACE2 and cyan denotes the SARS-CoV-2 spike protein in the figures of molecular docking.

## Discussion

Finding possible antiviral drugs against SARS-CoV-2 is extremely urgent with the rapid spread of COVID-19. However, it seems very difficult to design a novel drug for COVID-19 within a very short time. One of efficient ways is to identify new clues of the treatment from FDA-approved drugs.

In our proposed VDA-RWR method, we computed the association scores for each virus-drug pair to predict potential antiviral drugs against SARS-CoV-2 based on random walk with restart and biological information of viruses and drugs. The originality of our proposed VDA-RWR method remains, constructing three small datasets and inferring possible antiviral chemical agents against SARS-CoV-2 from FDA-approved drugs. The comparative experiments showed better performance of the VDA-RWR method. Higher AUC values under three fivefold CVs on three datasets and molecular binding energies indicated that the selected small molecules are likely to be used to stop the transmission of COVID-19.

VDA-RWR can obtain superior performance under the three fivefold CVs on three datasets. This observation may be attributed to random walk with restart, a state-of-the-art model that can randomly walk on the heterogeneous virus-drug network and effectively compute association score for each virus-drug pair. More importantly, VDA-RWR integrated various biological information including the complete genome sequences of viruses and chemical structures of chemical agents.

The proposed VDA-RWR method is also helpful in design and interpretation of pharmacological experiment related to COVID-19. More importantly, VDA-RWR can be further applied to predict antiviral drugs against novel viruses without any associated chemical agents.

## Methods

### Virus-drug association data

#### Dataset 1

##### Virus data

We considered 11 viruses similar to SARS-CoV-2. These viruses include influenza A viruses including A-H1N1^32^, A-H5N1^33^, and A-H7N9^34^, chronic hepatitis C virus (HCV)^35^, human immunodeficiency virus type 1 (HIV-1)^36^, human immunodeficiency virus type 2 (HIV-2)^37^, hendra virus^38^, human cytomegalovirus^39^, MERS-CoV^40^, respiratory syncytial virus^41^ and SARS-CoV^42^. The complete genome sequences of these viruses are downloaded from the NCBI database^43^. We used MAFFT^44^ (https://mafft.cbrc.jp/alignment/software/, version 7, open source license: GPL or BSD), a multiple sequence alignment tool, to compute virus-virus sequence similarity matrix ${S}_{v}$. All parameters were set as the default values provided by MAFFT.

##### Drug data

We manually curated drugs associated with these 11 viruses from the DrugBank^45^ and NCBI^43^ databases and published literatures reported by the PubMed database^46^ and collected 78 small molecules after removing macromolecules. Based on the assumption that two drugs are more similar if they share more chemical substructures, drug-drug similarity can be computed. Extended connectivity fingerprints (ECFPs)^47^ are circular fingerprints and developed for structure–activity modeling and molecular feature description. We used RDKit^48^ (https://github.com/rdkit/rdkit, releases 131, open source license: BSD), an open-source cheminformatics software, to compute ECFPs of drugs with a radius of 2. Drug-drug chemical structure similarity matrix ${S}_{d}$ can be computed by the ECFPs of drugs.

##### VDAs

We searched the publicly available repositories including the DrugBank^45^ and NCBI^43^ databases and publications reported by the PubMed database^46^. At the time of writing, we obtained 96 virus-drug associations (VDAs) between 11 viruses and 78 drugs. We described A-H1N1^32^, A-H5N1^33^, and A-H7N9^34^ as three viruses although they belong to influenza A for the sake of description.

#### Dataset 2

The DrugVirus.info database^49^ (https://drugvirus.info/) provided various VDA-related resources. We obtained 770 VDAs from 69 viruses and 128 drugs after removing the viruses whose complete genome sequences are unknown from the database. The chemical structure of drugs and the complete genome sequences of viruses were downloaded from the DrugBank database and the NCBI database, respectively. Similar to dataset 1, we used RDKit and MAFFT to calculate drug similarity and virus similarity.

#### Dataset 3

We retrieved 407 VDAs from 34 viruses and 203 drugs by searching documents related to viruses and drugs based on text mining techniques. Similar to dataset 1, we computed drug similarity matrix and virus similarity matrix. The details of three datasets are shown in Table 9.

**Table 9:** Statistics for the virus-drug association networks.

Datasets	Viruses	Drugs	VDAs
Dataset 1	12	78	96
Dataset 2	69	128	770
Dataset 3	34	203	407

In this study, the set of known VDAs was considered as the ‘gold standard’ dataset and was applied to evaluate the performance of our proposed VDA-RWR method. We described the known VDAs as a matrix $Y$:

7 $$Y_{ij} = \left\{ {\begin{array}{*{20}l} 1 \hfill & {if\;v_{i} \,associates\,with\;d_{j} } \hfill \\ 0 \hfill & {otherwise} \hfill \\ \end{array} } \right.$$

where ${v}_{i}$ and ${d}_{j}$ represent the $i$ th virus and $j$ th drug, respectively.

### The VDA-RWR method

**Figure 2:**
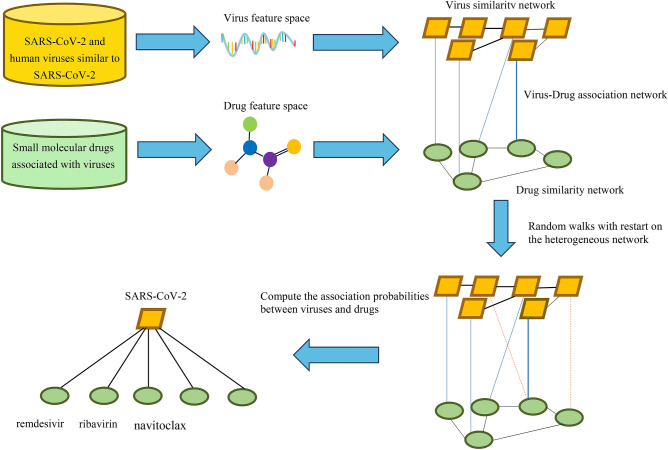
Flowchart of the VDA-RWR method based on the genome sequences of viruses, the chemical structures of drugs, and random walk with restart on the heterogeneous network.

Inspired by the method provided by Valdeolivas et al.^50^, we developed a VDA prediction method based on Random Walk with Restart on the heterogeneous network (VDA-RWR). The proposed VDA-RWR method comprised two steps. First, a random walk-based model integrating various biological data was learned to explain the constructed ‘gold standard’ dataset. Second, this model was used to find potential VDAs for viruses and drugs absent from the ‘gold standard’ dataset. The details are shown Fig. 2.

We first considered virus-virus similarity graph ${\mathrm{G}}_{\mathrm{v}}$, drug-drug similarity graph ${\mathrm{G}}_{\mathrm{d}}$, and VDA graph ${\mathrm{G}}_{\mathrm{a}}$, which formed a heterogeneous network. We defined ${\mathrm{S}}_{\mathrm{v}(\mathrm{n}\times \mathrm{n})}$, ${\mathrm{S}}_{\mathrm{d}(\mathrm{m}\times \mathrm{m})}$, and ${\mathrm{Y}}_{(\mathrm{n}\times \mathrm{m})}$ as their corresponding adjacency matrices. The adjacency matrix of the heterogeneous network can be denoted as: $$W=\left[\begin{array}{cc}{S}_{v}& Y\\ {Y}^{T}& {S}_{d}\end{array}\right]$$, where ${Y}^{T}$ denoted the transpose of the VDA matrix $Y$.

We then calculated the transition probabilities of random walk with restart on the heterogeneous network. Suppose $$W=\left[\begin{array}{cc}{W}_{vv}& {W}_{vd}\\ {W}_{dv}& {W}_{dd}\end{array}\right]$$ represented the matrix of transitions on the heterogeneous network, where ${W}_{vv}$/${W}_{dd}$ denoted the walk within the virus/drug network, ${W}_{vd}$/${W}_{dv}$ described the jump from the virus/drug network to the drug/virus network. Given the probability $\mu$ of jumping from the virus/drug network to the drug/virus network, the transition probability from virus ${v}_{i}$ to virus ${v}_{j}$ was defined as

8 $$W_{vv} \left( {i,j} \right) = \left\{ {\begin{array}{*{20}l} {\frac{{S_{v} \left( {i,j} \right)}}{{\mathop \sum \nolimits_{k = 1}^{n} S_{v} \left( {i,k} \right)}}} \hfill & {if\;\mathop \sum \limits_{k = 1}^{m} Y\left( {i,k} \right) = 0} \hfill \\ {\frac{{\left( {1 - \mu } \right)S_{v} \left( {i,j} \right)}}{{\mathop \sum \nolimits_{k = 1}^{n} S_{v} \left( {i,k} \right)}}} \hfill & {otherwise} \hfill \\ \end{array} } \right.$$

The transition probability from virus ${v}_{i}$ to drug ${d}_{j}$ was defined as

9 $$W_{vd} \left( {i,j} \right) = \left\{ {\begin{array}{*{20}l} {\frac{{\mu Y\left( {i,j} \right)}}{{\mathop \sum \nolimits_{k = 1}^{m} Y\left( {i,k} \right)}}} \hfill & {if\;\mathop \sum \limits_{k = 1}^{m} Y\left( {i,k} \right) \ne 0} \hfill \\ 0 \hfill & {otherwise} \hfill \\ \end{array} } \right.$$

The transition probability from drug $d_{i}$ to drug $d_{j}$ was defined as

10 $$W_{dd} \left( {i,j} \right) = \left\{ {\begin{array}{*{20}l} {\frac{{S_{d} \left( {i,j} \right)}}{{\mathop \sum \nolimits_{k = 1}^{m} S_{d} \left( {i,k} \right)}}} \hfill & {if\;\mathop \sum \limits_{k = 1}^{n} Y\left( {k,i} \right) = 0} \hfill \\ {\frac{{\left( {1 - \mu } \right)S_{d} \left( {i,j} \right)}}{{\mathop \sum \nolimits_{k = 1}^{m} S_{d} \left( {i,k} \right)}}} \hfill & {otherwise} \hfill \\ \end{array} } \right.$$

The transition probability from drug $d_{i}$ to virus $v_{j}$ was defined as

11 $$W_{dv} \left( {i,j} \right) = \left\{ {\begin{array}{*{20}l} {\frac{{\mu Y\left( {j,i} \right)}}{{\mathop \sum \nolimits_{k = 1}^{n} Y\left( {k,i} \right)}}} \hfill & { if\;\mathop \sum \limits_{k = 1}^{n} Y\left( {k,i} \right) \ne 0} \hfill \\ 0 \hfill & { otherwise} \hfill \\ \end{array} } \right.$$

For a given virus/drug, the particle can either jump between graphs or stay in the current graph with a defined probability $r\in \left(\mathrm{0,1}\right)$. Therefore, we finally defined the random walk with a restart probability $r$ as:

12 $${p}_{t+1}=rW{p}_{t}+\left(1-r\right){p}_{0}$$

where ${p}_{t}$ represented the computed association probability at the $t$-th step random walk. We defined the initial probability as: $${p}_{0}=\left[\begin{array}{c}\alpha {u}_{0}\\ \left(1-\alpha \right){t}_{0}\end{array}\right]$$, where ${u}_{0}$ and ${t}_{0}$ denoted the initial probability on the drug network and the virus network, respectively. If we tend to identify possible drugs for a given virus ${v}_{i}$, it is considered as a seed node in the virus network. Here, ${v}_{i}$ was assigned as 1 and other nodes as 0, constructing the initial probability of the virus network ${t}_{0}$. All nodes in the drug network ${u}_{0}$ were assigned as equal probabilities with the sum of 1. For example, to find potential antiviral drugs against SARS-CoV-2, we set SARS-CoV-2 as a seed node, and all drugs in the drug network were assigned as the same probabilities with the values of $1/m$. The parameter $\mathrm{\alpha }$ was used to control the weight of the virus network and the drug network. In addition, a virus is new if it does not associate with any drugs, and a drug is new if it is not applied to any viruses.

### Molecular docking

Molecular docking technique was applied to compute the intermolecular binding abilities between the predicted anti-SARS-CoV-2 drugs and the SARS-CoV-2 spike protein/human ACE2. The chemical structures of drugs were downloaded in the form of the PDB format files from the DrugBank database. We used AutoDockTools to convert these PDB files into pdbqt files needed by AutoDock4. The structures of SARS-CoV-2 spike receptor-binding domain bound with ACE2 (PDB ID: 6M0J) were downloaded from the RCSB Protein Data Bank^51^. The spike protein and ACE2 were used as receptors, and the predicted anti-SARS-CoV-2 drugs were used as ligands for the molecular docking.

We first removed solvent and organic compounds and preprocessed the receptor proteins based on PyMOL^31^ (https://github.com/schrodinger/pymol-open-source, release 2.4.0, open source license: BSD-like). The receptors’ atoms were assigned the AD4 type and Gasteiger charges were considered before docking. Molecular docking software, AutoDock^19^ (http://autodock.scripps.edu/, AutoDock 4.2.6, open source license: GPL), was then used to conduct molecular docking. The binding pocket was defined by AutoGrid4, the grid size was set to 82 × 154 × 84 with a spacing of 0.375 Å, and the grid center was placed at the area of SARS-CoV-2 spike receptor-binding domain bounding with ACE2 (x = − 36.884, y = 29.245, z = − 0.005). The LGA (Lamarckian genetic algorithm) with default parameter provided by AutoDock4 was used as the search method. The docking contained two main processes: computation of conformation, position, and orientation of ligands within the binding sites and ranking of these conformations based on the binding affinities^18^.

## Conclusion

To find potential antiviral drugs, in this study, we integrated the complete genome sequences of viruses, the chemical structures of drugs, and the VDA network. We then developed a VDA prediction method based on random walk with restart on the heterogeneous network. The results suggested that remdesivir and ribavirin may be applied to the treatment of COVID-19. In the emergency situation, this study focused more on finding antiviral drugs. In the future, we will further integrate more biological data and design more powerful models to improve the accuracy of VDA identification. We hope that our proposed VDA-RWR method could help the screening of drugs for preventing COVID-19.

## Supplementary Information

Supplementary Information 1.

Supplementary Information 2.

Supplementary Information 3.

## Data Availability

Source codes and datasets are freely available for download at https://github.com/plhhnu/VDA-RWR/.
