# Supplementary figures and images for: Prioritizing antiviral drugs against SARS-CoV-2 by integrating viral complete genome sequences and drug chemical structures
Source: Sci Rep. 2021 Mar 18;11:6248. doi: 10.1038/s41598-021-83737-5 (PMC7973547; doi:10.1038/s41598-021-83737-5)

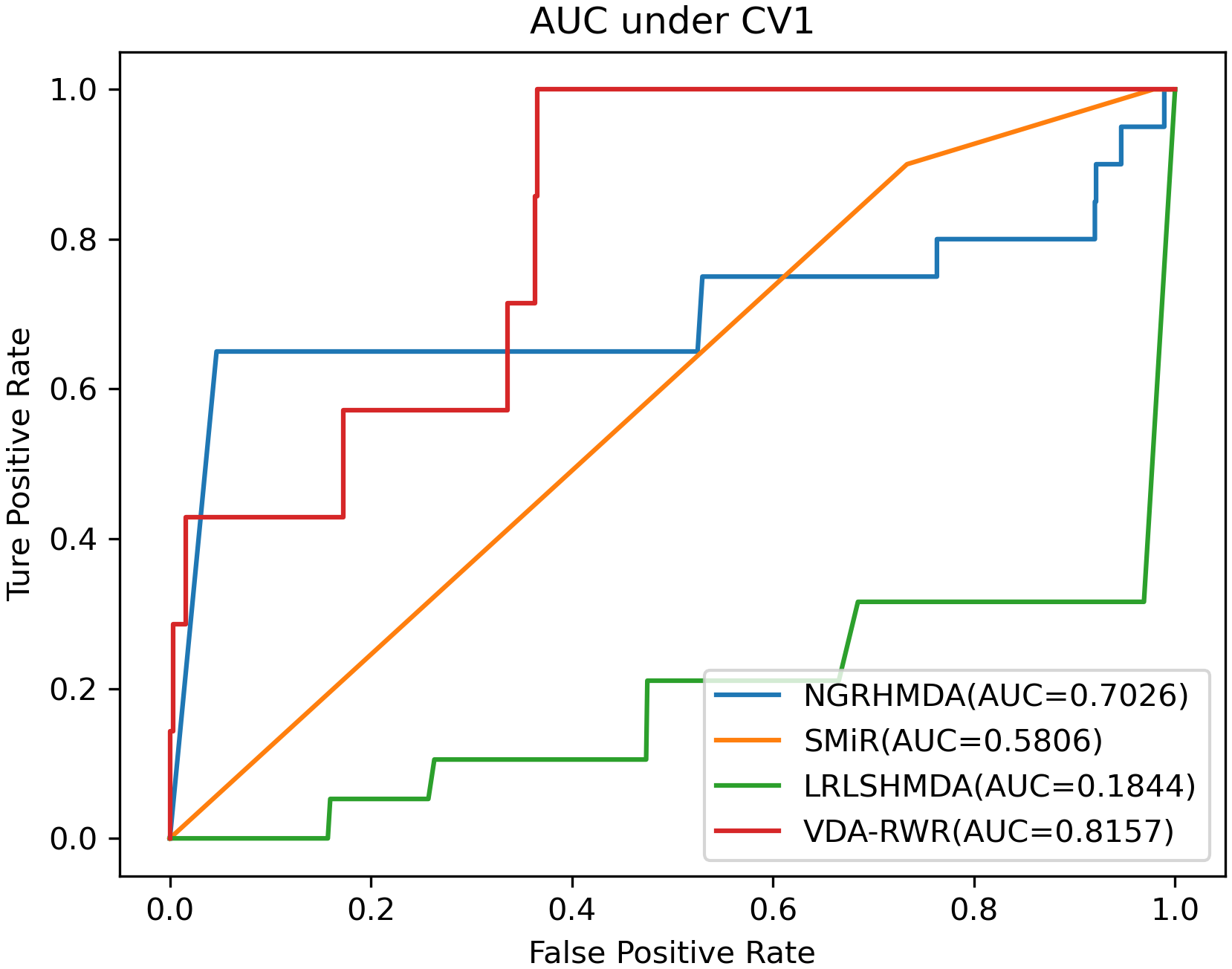

Supplement: Supplementary file 2 — Supplementary Information 2. [file 41598_2021_83737_MOESM2_ESM.zip › figures_AUC_VDA-RWR/dataset1/cv1.png]

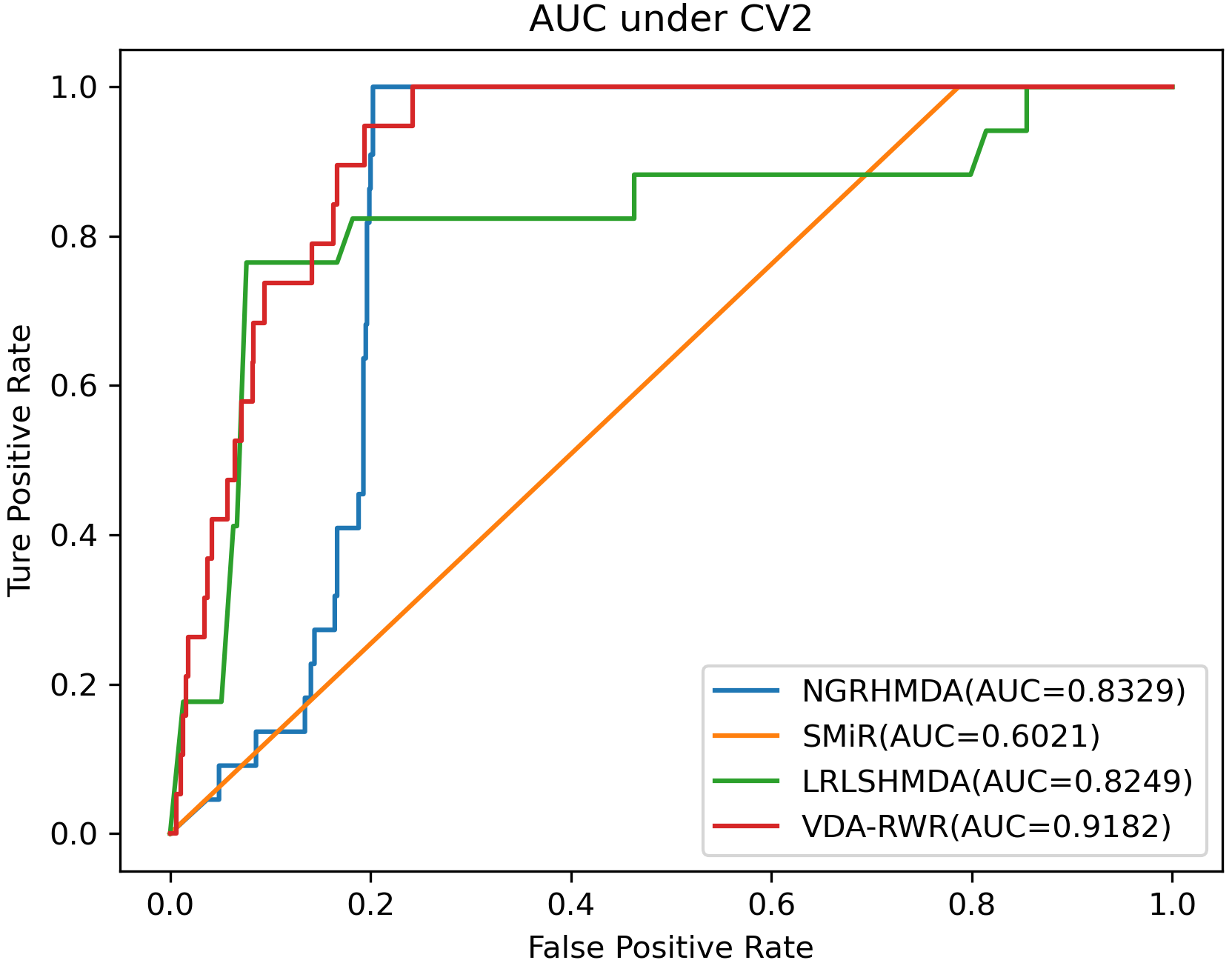

Supplement: Supplementary file 2 — Supplementary Information 2. [file 41598_2021_83737_MOESM2_ESM.zip › figures_AUC_VDA-RWR/dataset1/cv2.png]

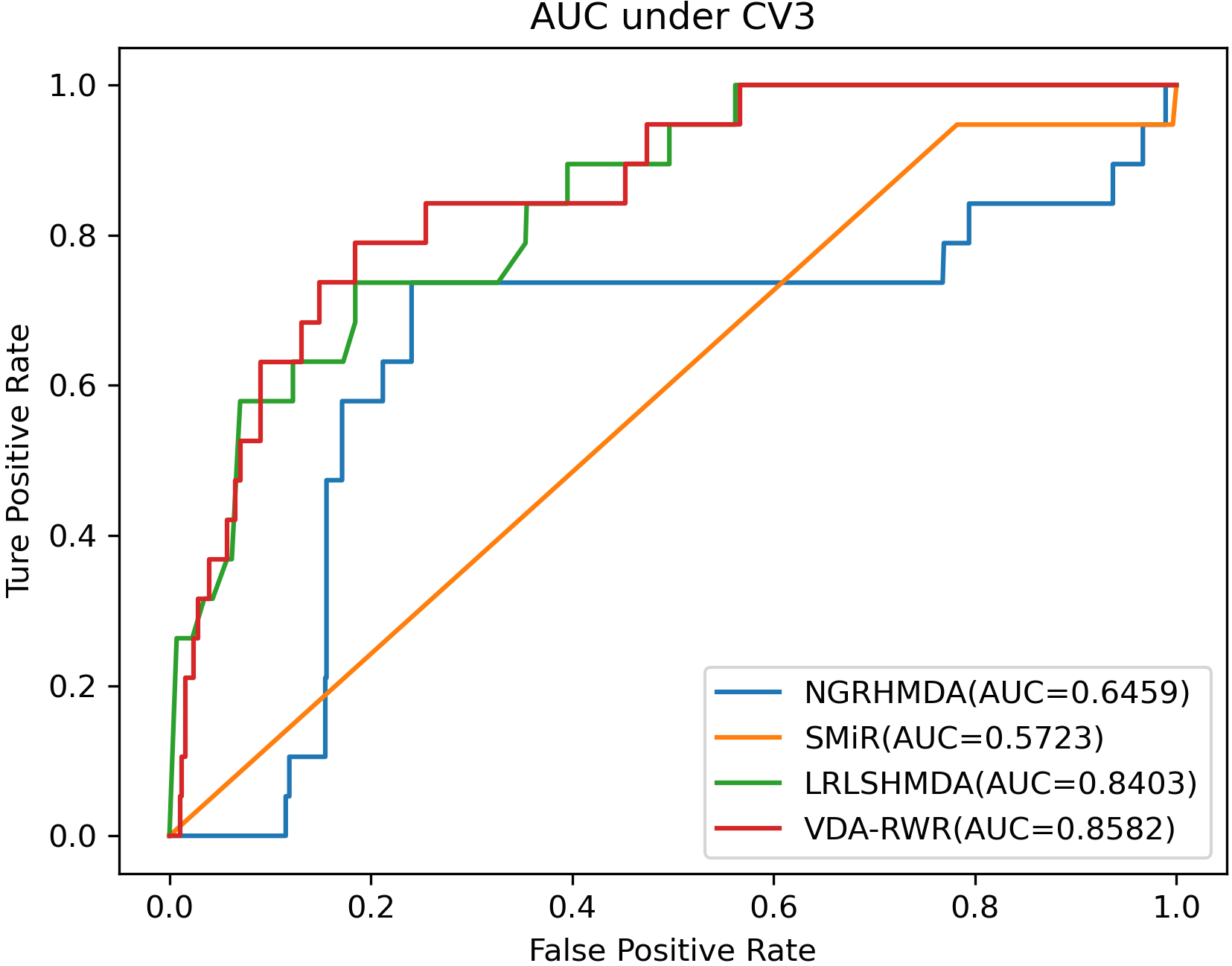

Supplement: Supplementary file 2 — Supplementary Information 2. [file 41598_2021_83737_MOESM2_ESM.zip › figures_AUC_VDA-RWR/dataset1/cv3.png]

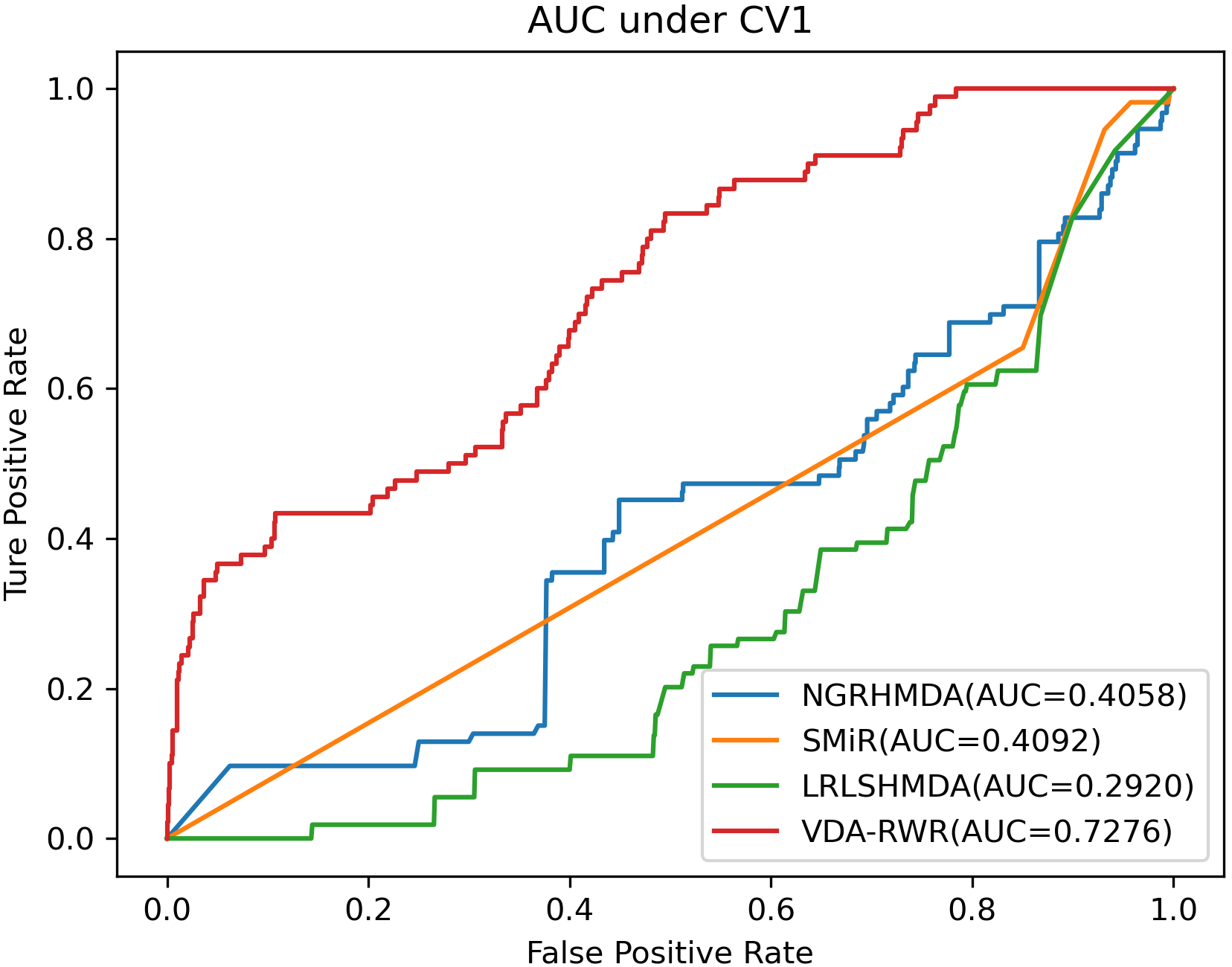

Supplement: Supplementary file 2 — Supplementary Information 2. [file 41598_2021_83737_MOESM2_ESM.zip › figures_AUC_VDA-RWR/dataset2/cv1.png]

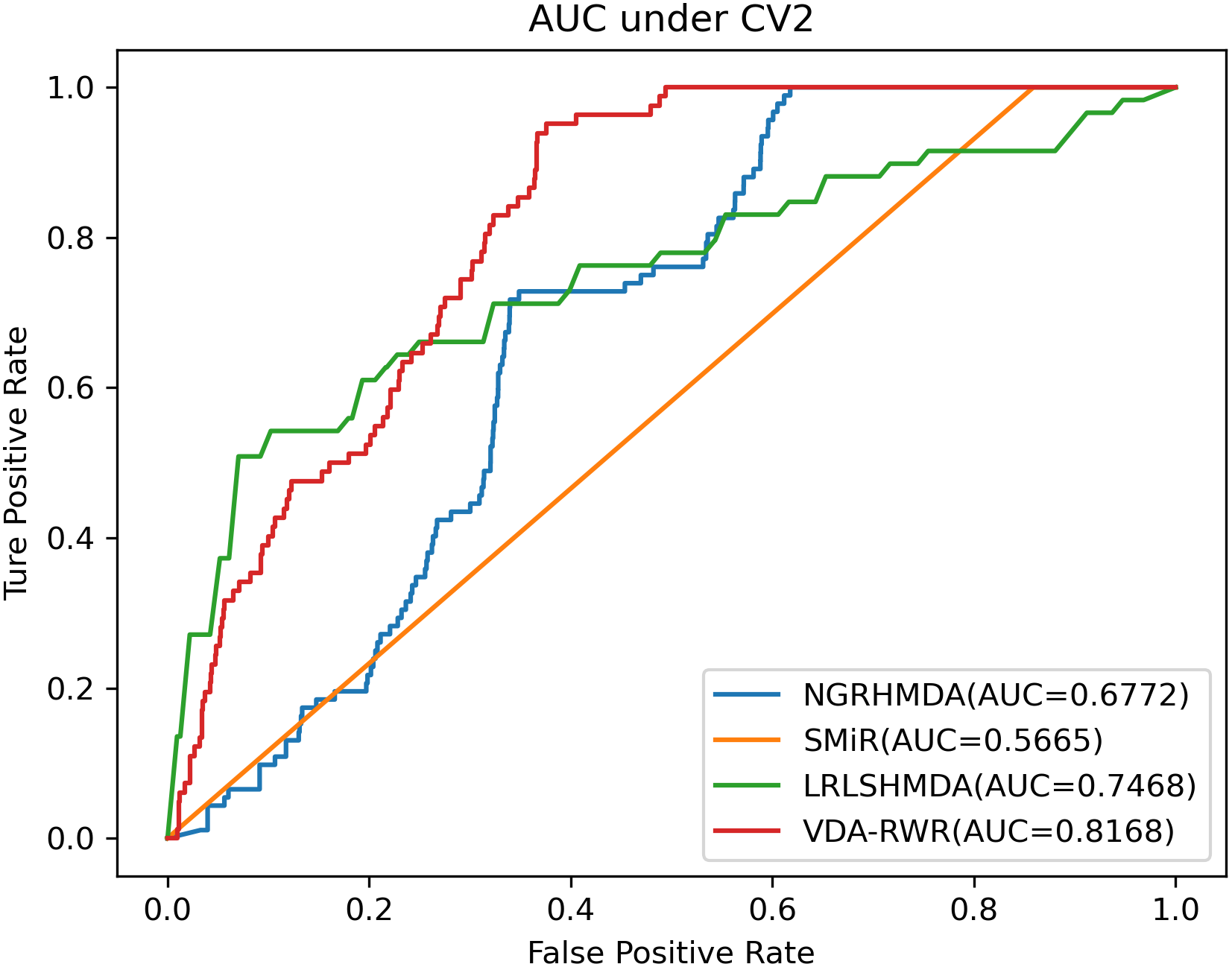

Supplement: Supplementary file 2 — Supplementary Information 2. [file 41598_2021_83737_MOESM2_ESM.zip › figures_AUC_VDA-RWR/dataset2/cv2.png]

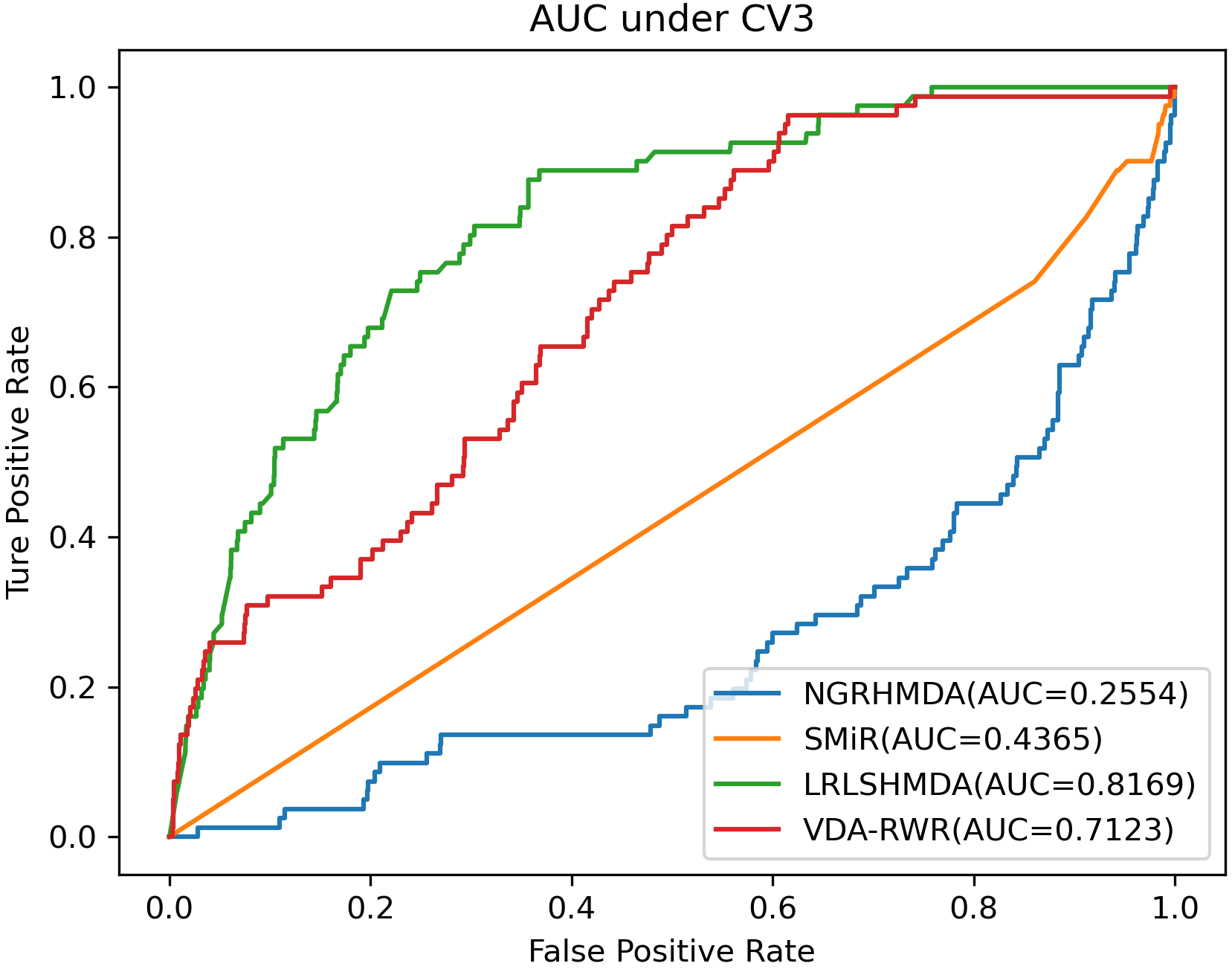

Supplement: Supplementary file 2 — Supplementary Information 2. [file 41598_2021_83737_MOESM2_ESM.zip › figures_AUC_VDA-RWR/dataset2/cv3.png]

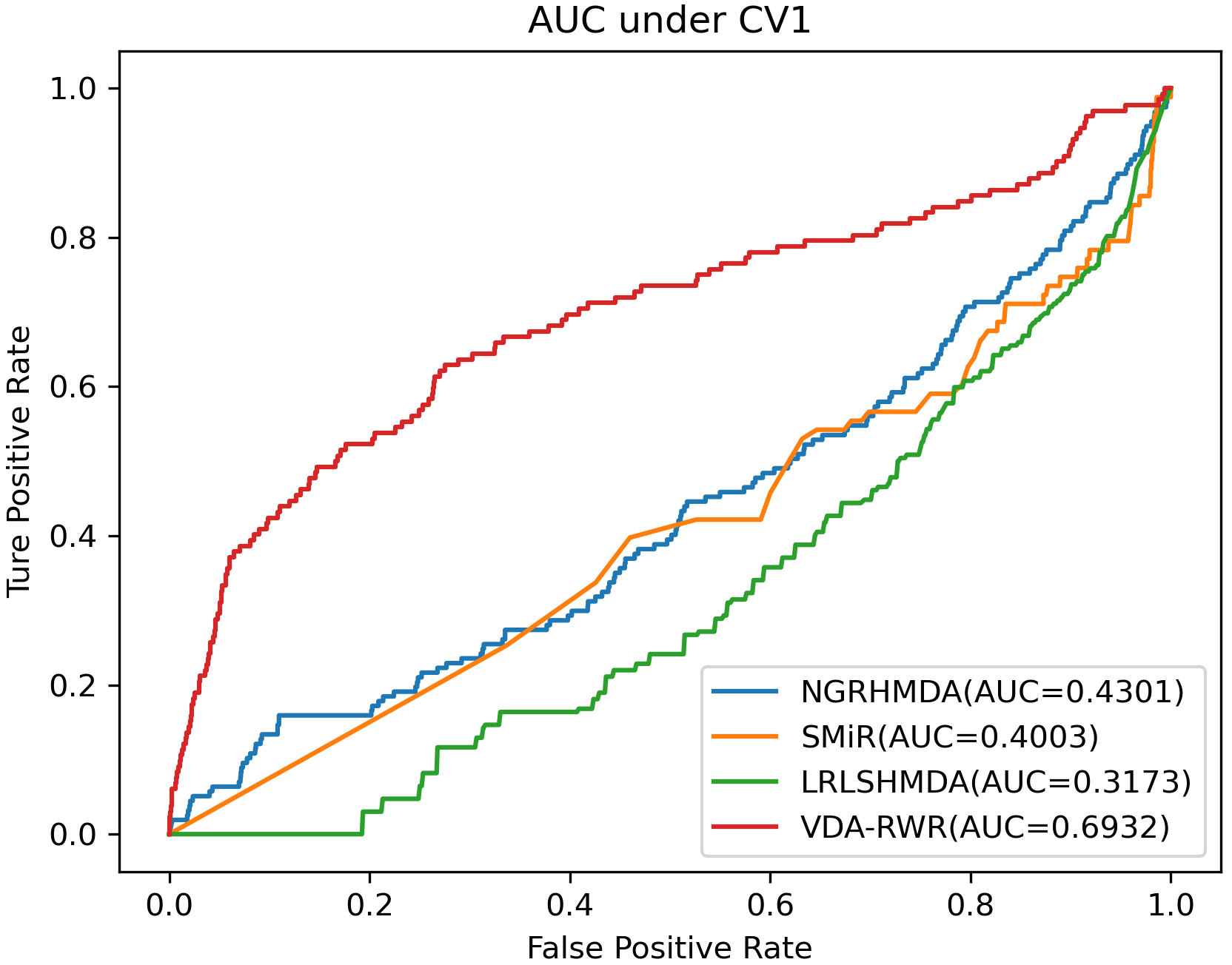

Supplement: Supplementary file 2 — Supplementary Information 2. [file 41598_2021_83737_MOESM2_ESM.zip › figures_AUC_VDA-RWR/dataset3/cv1.png]

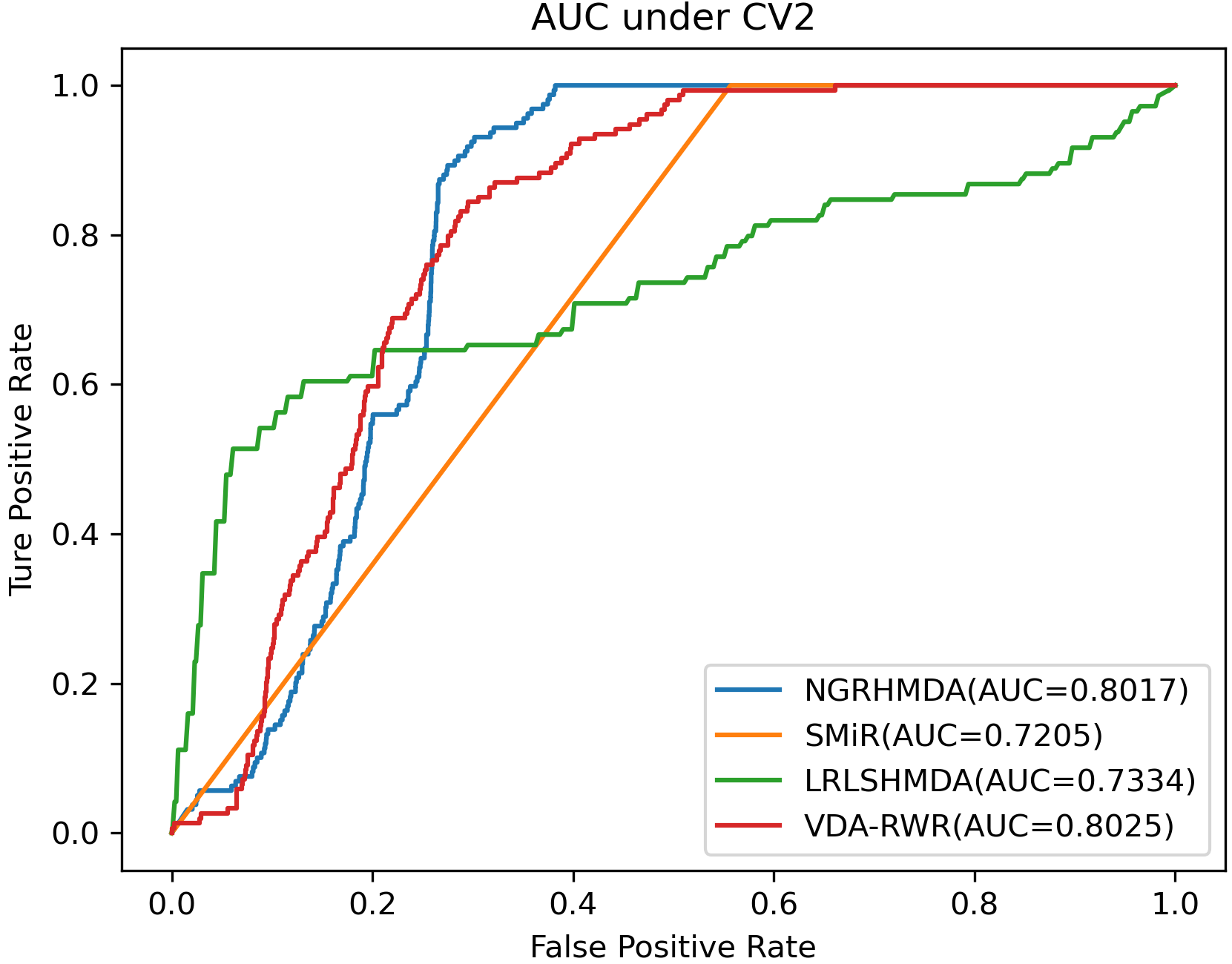

Supplement: Supplementary file 2 — Supplementary Information 2. [file 41598_2021_83737_MOESM2_ESM.zip › figures_AUC_VDA-RWR/dataset3/cv2.png]

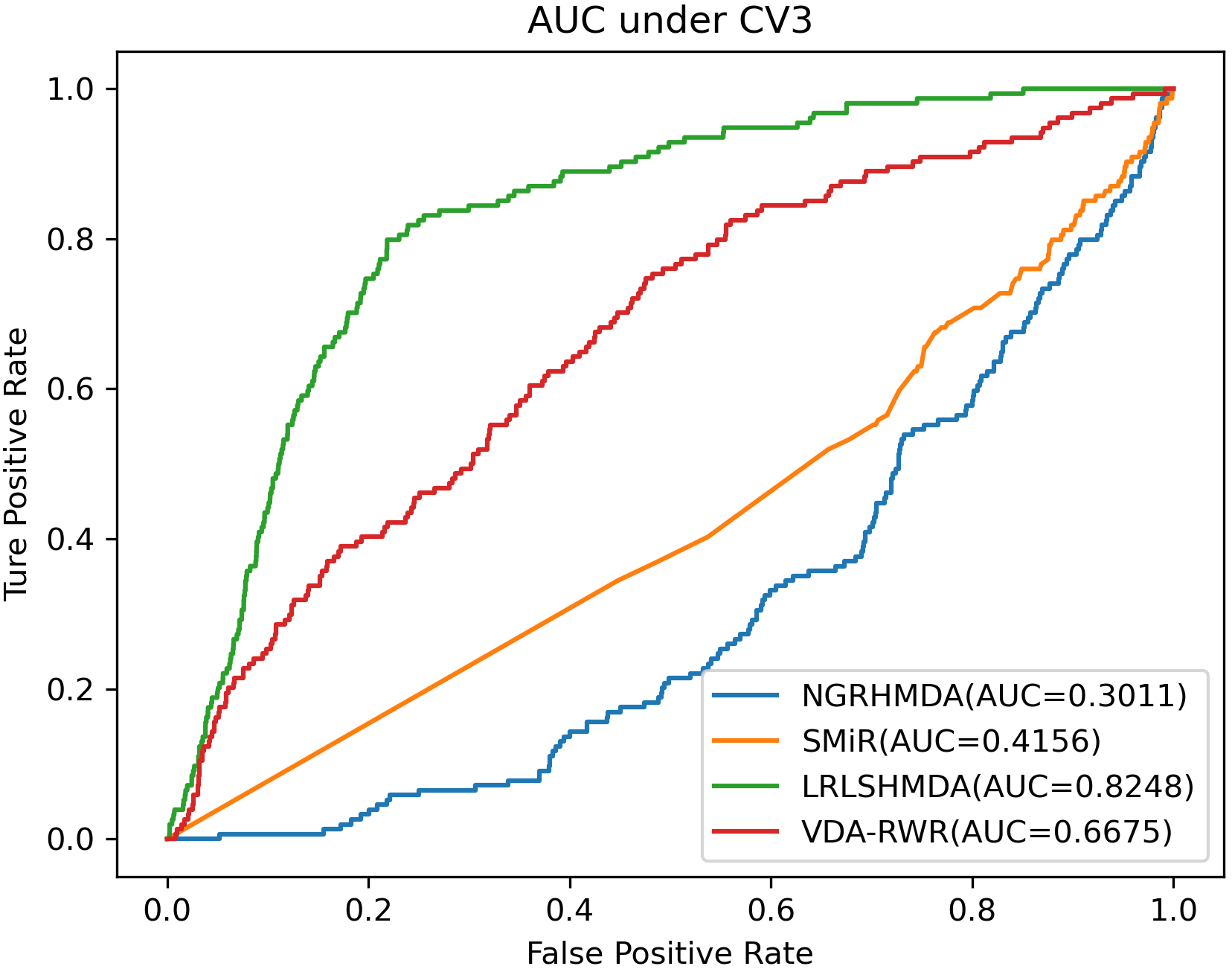

Supplement: Supplementary file 2 — Supplementary Information 2. [file 41598_2021_83737_MOESM2_ESM.zip › figures_AUC_VDA-RWR/dataset3/cv3.png]

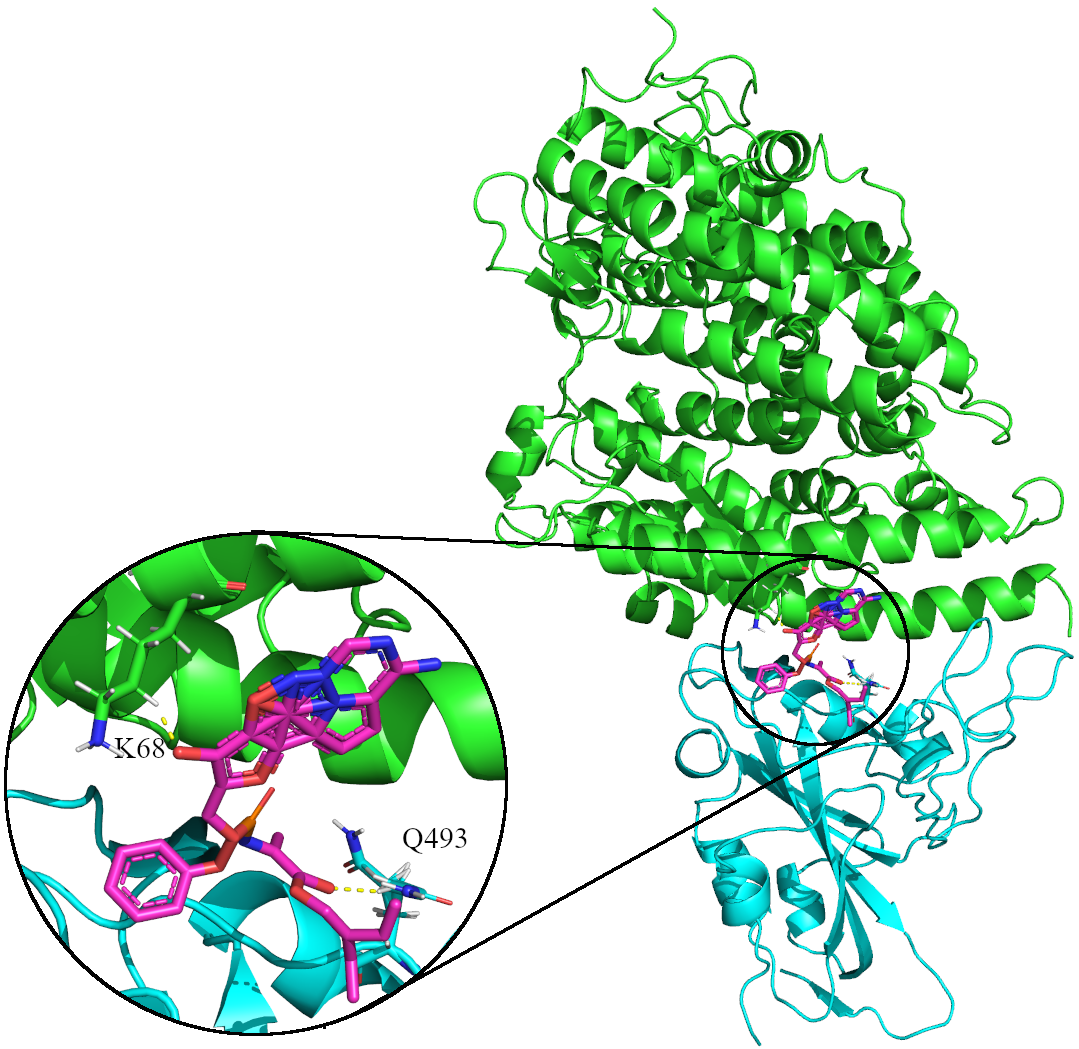

Supplement: Supplementary file 3 — Supplementary Information 3. [file 41598_2021_83737_MOESM3_ESM.zip › docking_figures+figure 2_VDA-RWR/docking_remdesivir.png]

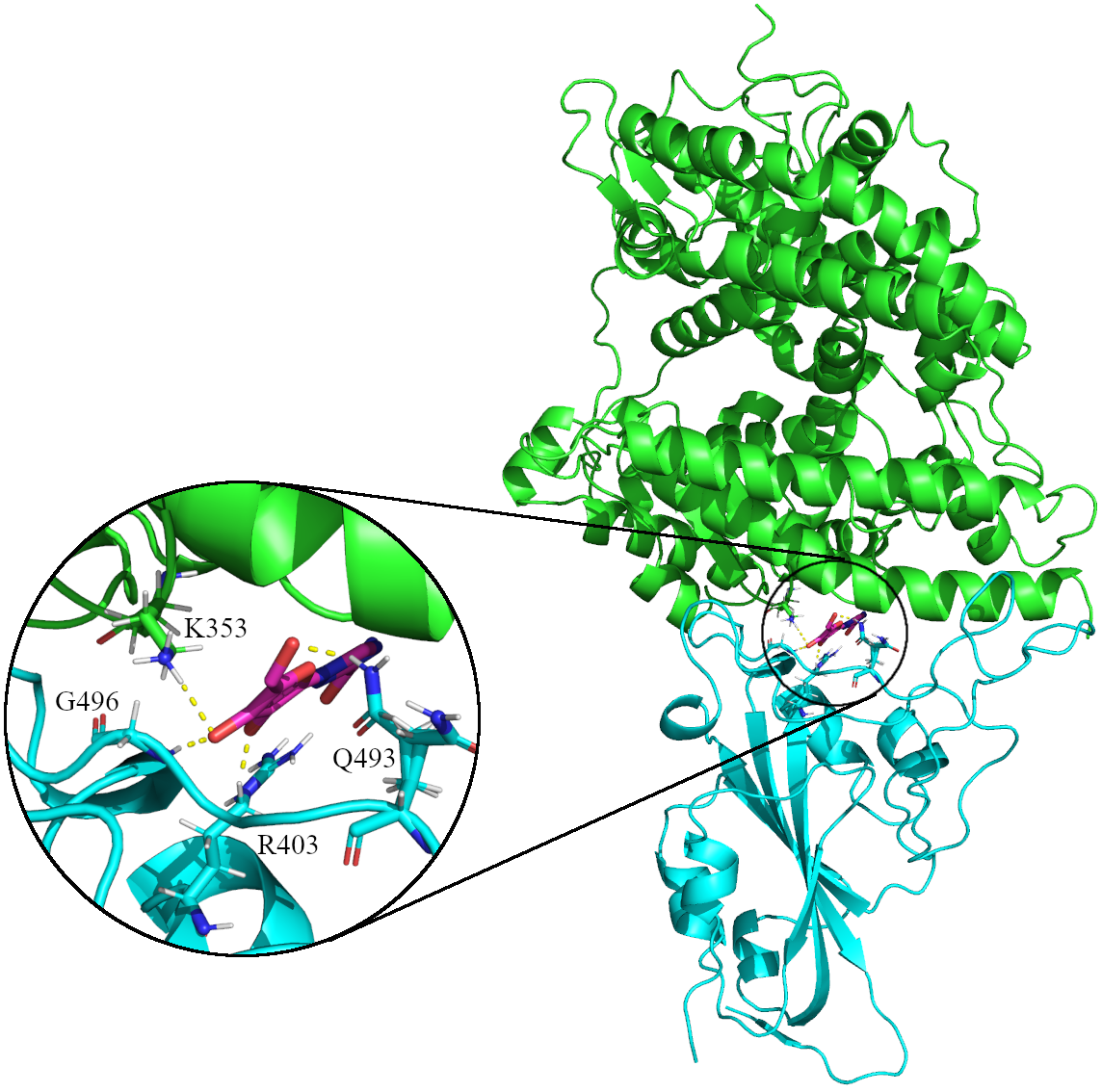

Supplement: Supplementary file 3 — Supplementary Information 3. [file 41598_2021_83737_MOESM3_ESM.zip › docking_figures+figure 2_VDA-RWR/docking_ribavirin.png]

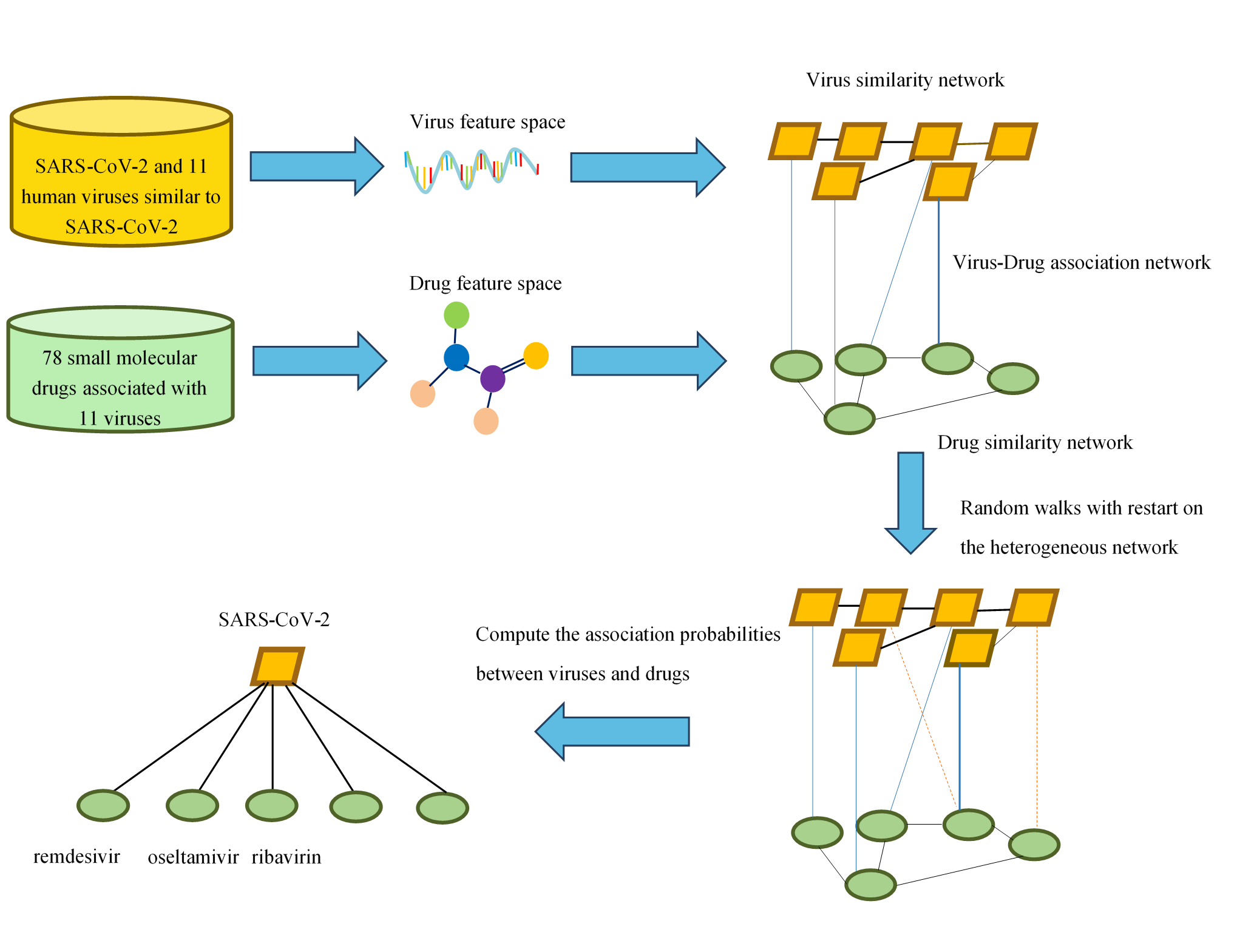

Supplement: Supplementary file 3 — Supplementary Information 3. [file 41598_2021_83737_MOESM3_ESM.zip › docking_figures+figure 2_VDA-RWR/Figure 2.tif]
