# Supplementary material for: Prioritizing antiviral drugs against SARS-CoV-2 by integrating viral complete genome sequences and drug chemical structures
Source: Sci Rep. 2021 Mar 18;11:6248. doi: 10.1038/s41598-021-83737-5 (PMC7973547; doi:10.1038/s41598-021-83737-5)
Supplement: Supplementary file 3 — Supplementary Information 3. [file 41598_2021_83737_MOESM3_ESM.zip › docking_figures+figure 2_VDA-RWR/readme_docking_figures+figure 2_VDA-RWR.docx]

Prioritizing antiviral drugs against SARS-CoV-2 by integrating viral complete genome sequences and drug chemical structures

Lihong Peng ^1,+^, Ling Shen ^1,+^, Junlin Xu ^2^, Xiongfei Tian ^1^, Fuxing Liu ^1^, Juanjuan Wang ^1^, Geng Tian ^2^, Jialiang Yang ^2,*^, Liqian Zhou ^1,*^

^1^School of Computer Science, Hunan University of Technology, Zhuzhou, 412007, China;

^2^College of Computer Science and Electronic Engineering, Hunan University, Changsha, 410082, China

^3^Geneis (Beijing) Co. Ltd., Beijing, 100102, China;

***Correspondence:**
J.L.Y.

yangjl@geneis.cn

L.Q.Z.

zhoulq11@163.com

^+^**these authors contributed equally to this work**

Abstract

The outbreak of a novel febrile respiratory disease called COVID-19, caused by a newfound coronavirus SARS-CoV-2, has brought a worldwide attention. Prioritizing approved drugs is critical for quick clinical trials against COVID-19. In this study, we first manually curated three Virus-Drug Association (VDA) datasets. By incorporating VDAs with the similarity between drugs and that between viruses, we constructed a heterogeneous Virus-Drug network. A novel Random Walk with Restart method (VDA-RWR) was then developed to identify possible VDAs related to SARS-CoV-2. We compared VDA-RWR with three state-of-the-art association prediction models based on 5-fold cross-validations (CVs) on viruses, drugs and virus-drug associations on three datasets. VDA-RWR obtained the best AUCs for the three 5-fold CVs, significantly outperforming other methods. We found two small molecules coming together on the three datasets, that is, remdesivir and ribavirin. These two chemical agents have higher molecular binding energies of -7.0 kcal/mol with human receptor angiotensin converting enzyme 2 (ACE2) and -6.59 kcal/mol with the SARS-CoV-2 spike protein, respectively. Interestingly, for the first time, experimental results suggested that navitoclax could be potentially applied to stop SARS-CoV-2 and remains to further validation.

Folder “docking_figures+figure 2_VDA-RWR” includes:

- The molecular docking figures between two predicted small molecules including remdesivir and ribavirin and SARS-CoV-2 spike receptor-binding domain bound with ACE2.
- Figure 2.
